# Ultrasonic Processing of Aluminum–Magnesium Alloys

**DOI:** 10.3390/ma11101994

**Published:** 2018-10-16

**Authors:** Kurt Mills, Gui Wang, David StJohn, Matthew Dargusch

**Affiliations:** 1 Defence Materials Technology Centre (DMTC), The University of Queensland, St Lucia QLD 4072, Australia; gui.wang@uq.edu.au (G.W.); d.stjohn@uq.edu.au (D.S.); m.dargusch@uq.edu.au (M.D.); 2 Centre for Advanced Materials Processing and Manufacturing, School of Mechanical and Mining Engineering, The University of Queensland, St Lucia QLD 4072, Australia

**Keywords:** ultrasonic treatment, aluminum, magnesium, alloy, grain refinement, solute, nucleation, microstructure, solidification

## Abstract

This study evaluated the effect of UltraSonic Treatment (UST) on a range of Al–Mg alloys. Previous research was carried out on single magnesium compositions. However, the amount and type of the alloy addition are known to affect the grain size even under UST, and the aim of this study was to determine whether or not alloy composition plays a similar role in the case of Al–Mg alloys. By testing binary Al–Mg alloys cast under regular casting conditions and under the presence of an ultrasonic field, it was found that while the addition of Mg solute is important, the amount of solute has little effect when UST is applied. It was observed that the grain size was barely affected by extra solute additions in this condition. This is due to the application of UST during solidification, which resulted in a dramatic reduction in the size of the nucleation free zone thus promoting many more successful nucleation events. Acoustic streaming is proposed as the main cause of this reduction in grain size.

## 1. Introduction

Al–Mg alloys are known to have the highest strengths of all Al alloys without heat treatment, along with having good corrosion resistance and weld ability, resulting in the wide use of these alloys in industry (especially naval sectors). These include the 5000 series of wrought alloys, and the 500 series of cast alloys [1]. While Al–Mg alloys seem to be relatively resistant to hot tearing compared to other Al alloys, they still show a tendency to form micro-cracks [2] and porosity with increased Mg content due to an increase in entrapped Hydrogen [3]. The cast ability of the 500 series is relatively poor, especially when compared to other alloy series such as the 300 series Al–Si alloys [4]. A conventional process for improving the as-cast mechanical properties of metal castings is grain refinement [5,6], which also provides a uniform refined grain structure throughout the casting [7]. When grain refinement has been applied, a correlation between the grain size reduction, and the number of casting defects has been observed. These defects include: Porosity, macro-segregation, non-uniform properties, and hot and cold tearing [8,9]. Grain refinement of other alloy systems usually results in fewer defects and a reduction in hot tearing susceptibility. Reducing the number and size of these defects can lead to savings in time and money in the downstream processing of the castings. However, there is a lack of information regarding the effects of alloy chemistry and grain refinement techniques on these Al–Mg alloys.

### Theory

Changes in alloy composition are a common way to achieve grain refinement by using magnesium [10,11], as well as other alloying materials or master alloys [12,13,14,15]. Changes in the alloy composition can alter the grain size in two ways, by increasing the nucleation rate, and also by causing a decreased rate of crystallization. It should be noted however that these two effects can be interconnected, as the reduction of the rate of grain growth results in a faster rate of increased super-cooling which allows the earlier activation of nucleants already present in the melt that were previously unable to nucleate. These effects are often characterized by evaluating the Growth Restriction Factor, *Q* [16,17]. Here *Q* is defined as:

(1) $$\;Q=\;C_{0}m_{l}\left(k-1\right)\;$$

where *C*_0_ is the solute weight percentage, *m_l_* is the gradient of the liquids taken from the Al–Mg phase diagram, while *k* is the equilibrium partition coefficient. An approximately linear relationship can then be formed between the grain size and 1/*Q*, which takes the form:

(2) $$\;d=a+\frac{b}{Q}\;$$

where *d* is the average grain size, *a* is a constant based on the nuclei density in the melt, and represents the fraction of nucleation sites which become active, and *b* is another constant relating to the thermodynamic and chemical properties of the casting process [12,16]. While this relationship is useful in interpreting results, further information can be obtained from The Interdependence Model [18], which for UltraSonic Treatment (UST) takes the form:

(3) $$\;d=\frac{1}{\sqrt[{3}]{f\left(A\right)N_{v}}}+5.6\left(\frac{Dz\Delta T_{n}}{vQ}\right)\;$$

where *N_v_* is the number density of nucleation particles, and *f* is a measure of the fraction of nuclei that become active, which is a function of the ultrasonic intensity A. *D* and *v* are the diffusion coefficient and solid–liquid interface growth rate, while *z*∆*T_n_* is the incremental undercooling required for nucleation and is dependent on the thermal gradient in the melt [18].

**Figure 1:**
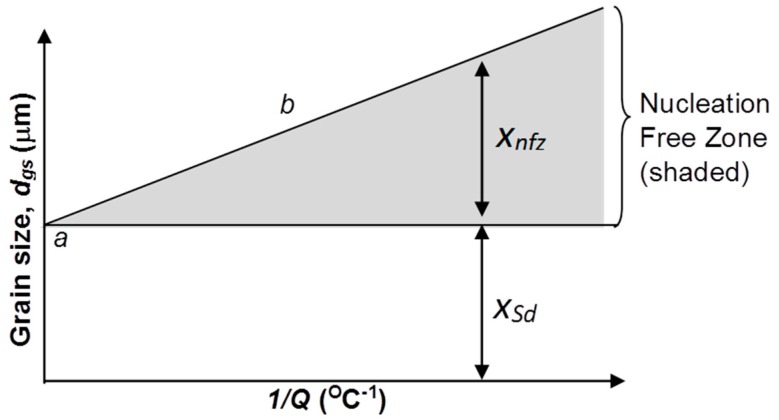
Illustration of the constants *a* and *b* where the horizontal line assumes a constant number density of nucleant particles. Adapted from [16].

This concept has been illustrated in a previous study [18] and shown in Figure 1. The constant *a* (also referred to as *x_Sd_*) is defined by the y-intercept. This value is related to the number of nuclei in the melt that can be activated and grow into grains, and it can be used as a measure of the minimum achievable grain size. The shaded section enclosed by the gradient, *b*, and *x_nfz_*, is referred to as the nucleation free zone (NFZ), a region containing potential nucleants that cannot nucleate due to insufficient constitutional super-cooling required to trigger nucleation in this zone.

In particular, certain inoculant and alloy additions have been found particularly useful and are termed grain refining master alloys. The most commonly used master alloys for Al alloys are Al–Ti–B master alloys, as they contain TiB_2_ intermetallic particles that act as nucleation sites, along with the growth restriction properties of Ti [13,19,20,21]. Other alloying elements have been found to have similar effects in Al alloys such as Si, Mg, and Cu [10,14,21].

Alternative methods of grain refinement have also been explored using external fields, such as mechanically-induced and electromagnetic fields [22,23], and the use of ultrasonics. Some studies have applied UST to single Al–Mg compositions [24,25], as well as many other Al alloys [26,27,28,29].

While UST generates cavitation in the melt [22,30], the exact mechanisms of grain refinement still are not agreed upon. The three most commonly accepted mechanisms are undercooling of the melt due to bubble collapse, undercooling of bubble surface due to expansion, and dendrite fragmentation due to shockwaves from bubble collapse [22,31,32]. Along with these, UST also causes acoustic streaming of the melt, degassing, and de-agglomeration of inclusions, which likely influence the resultant grain structure [22,28,30,32].

All previous studies of the effect of UST on Al–Mg alloys only investigated a single Mg composition [29,33], rather than investigating the effects of UST over a range of alloy compositions. It is now well established that composition can have a significant effect on grain size when subjected to UST [27,28,33,34,35]. In these studies the effect of composition has been evaluated by relating grain size to the inverse of *Q* (i.e., 1/*Q*). An aim of this study is to determine whether or not the same relationships with *Q* previously observed are developed when UST is applied to a range of Al-Mg alloys. The results of this study allow comparison of the grain sizes produced by UST and without UST for a range of solute Mg additions. The ability to quantify the combined influence of solute addition, and the application of UST during the nucleation phase of solidification could lead to a reduction in defect size and improved mechanical performance.

## 2. Materials and Methods 

Four individual alloys (Al-5, 10, 15 and 20 wt % Mg) were cast for the experiments. Commercially pure base metals were used to make the alloys each with a purity of 99.7%, and cast in 3 kg batches after being melted in a 3.6-kW electric furnace. These bulk alloys were made to maintain consistency of composition for both regular casting and UST experiments.

Each sample was made by placing 300–350 g of the alloy in a clay-graphite crucible, with dimensions: 60 mm and 40 mm for the top and bottom diameters respectively, and a height of 80 mm. A single thermocouple (GPA-K K-type, Pyrosales, Sydney, Australia) was placed in the melt during cooling to generate thermal analysis plots. This was placed to one side of the crucible so as to be between the crucible wall and sonotrode (630-0617, Sonics, Newtown, CT, USA) during UST and was inserted to a depth of 40 mm. The ultrasonic waves were produced by a commercial ultrasonic generator (Sonics VCX1500, Sonics, Newtown, CT, USA) rated to 2-kW, with a 20-kHz piezoelectric transducer which was air-cooled. A 20 mm diameter titanium alloy sonotrode was used to transmit the ultrasound at an amplitude of 20 μm into the melt.

Once each specific binary alloy was prepared in the crucible, they were placed in an electric furnace produced in house at The Univerisity of Queenslandand heated to approximately 60 °C above the liquidus temperature for that binary mixture, as taken from the binary Al–Mg phase diagram. After being held at this temperature for a few minutes, the oxidised skin was removed from the sample’s surface, then it was transferred to an external platform for air cooling, and the thermocouple was inserted. As-cast samples without UST were then allowed to cool naturally in air. For UST samples, the melt was allowed to cool until the superheat was decreased to 40 °C, then the un-preheated sonotrode was inserted approximately 20 mm into the melt and switched on and UST was applied for 90 s. This range of time over which UST was applied was chosen based on a previous study where it was found that preheating was a key factor in preventing solidification around the sonotrode that would reduce the effectiveness of UST [28]. Once 90 s was reached, the sonotrode was switched off, and raised from the melt, allowing natural air cooling to resume.

After each sample had been cast, metallographic samples were produced from these castings by cutting each sample down the symmetrical axis. One-half was ground and polished, before being treated with a Tucker Etchant produced in house, with the concentration adjusted for each binary alloy, to reveal the macrostructure. Further samples were taken from the remaining halves, which were cut into approximately 15 mm^2^ samples, that were again ground and polished, though to a much finer scale. A Jeol JSM-6610 Scanning Electron Microscope (SEM, Jeol JSM-6610, Jeol Ltf, Sydney, Australia) was used to perform Electron Backscatter Diffraction (EBSD, Jeol JSM-6610, Jeol Ltf, Sydney, Australia), using the integrated Oxford/HKL EBSD detector. EBSD was employed to generate micrographic images for grain size analysis. The ASTM E112-10 standard [36] was used to calculate the average grain diameter, which was done using the linear intercept method. For each alloy, the *Q* value, was determined from the Al-Mg binary phase diagram, which was generated using the software package ThermoCalc (3.0.1, Thermo-Calc Software, Solna, Sweden).

## 3. Results and Discussion

**Figure 2:**
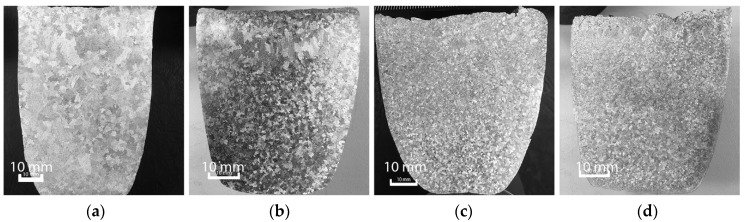
Macrostructures of (**a**) Al–5Mg; (**b**) Al–10Mg; (**c**) Al–15Mg; (**d**) Al–20Mg without the application of UST.

The macrostructure of all alloys formed under as-cast conditions can be seen in Figure 2. The as-cast Al-5Mg alloy macrostructure shown in Figure 2a, has large coarse and irregular grains. By increasing the Mg content to Al-10Mg, the reduction in grain size is evident, and the structure is much more uniform as shown in Figure 2b. This trend continues as the magnesium content increases to 20%, though the rate at which the grain size decreases appears to become slower as additional Mg is added. Figure 2c,d show this effect in more detail.

As seen in Figure 2, the grain structure becomes increasingly uniform with the addition of Mg to the alloys. However, a band of larger grains is observed along the top edge of most samples, which is due to these sections being exposed to air cooling during solidification. 

**Figure 3:**
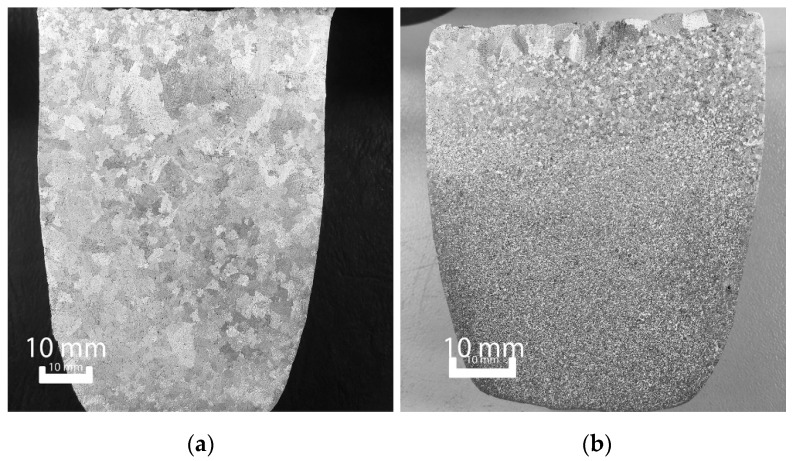
Macrostructure comparison (**a**) without and (**b**) with UST applied to the Al–5Mg

However, as shown in Figure 3, UST provides much greater grain refinement than the addition of Mg solute alone. For the same Al–5Mg alloy, UST results in a much smaller grain size, which is beyond the resolution of the macroscale. Figure 3b, however, shows that the effect of UST is mostly uniform throughout the ingot, though once again there is slight coarsening at the top of the casting. Additionally, either side of the sonotrode insertion point is slightly coarser, as UST cannot effectively treat this area.

**Figure 4:**
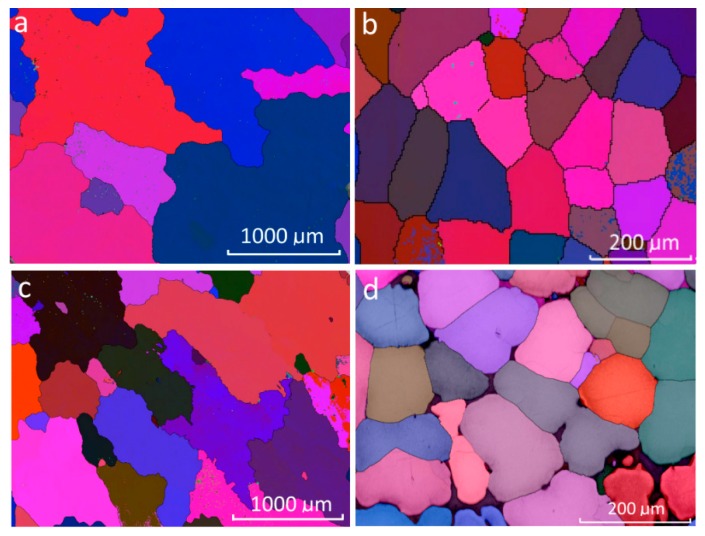
Microstructures of (**a**) Al–5Mg; (**b**) Al–5Mg w/UST; (**c**) Al–10Mg; (**d**) Al–10Mg w/UST.

To better observe the impact of UST on grain size, an analysis of the microstructure is required. By using the same alloys as an example, the dramatic refinement of the microstructure due to UST is observed in Figure 4. Note the difference in scale between the samples with and without UST. Some minor defects from the casting process are also evident in these images (particularly Figure 4d due to its higher resolution), which are attributed to porosity in the samples.

While the decrease in grain size between the Al–5Mg and Al–10Mg as-cast samples is still apparent (despite their average grain size being on the same scale as the EBSD resolution), the reduction caused by the application of UST is clear. Here, the individual grains can be clearly observed due to their different colors (which is due to the different grain orientations detected by the EBSD process), and the average grain size determined.

**Figure 5:**
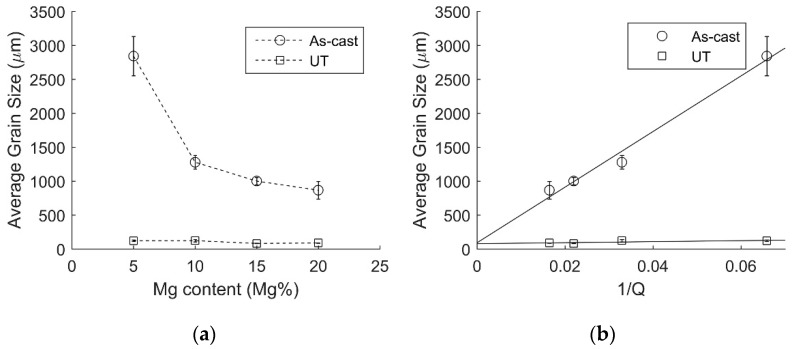
Plots of the average grain size (**a**) versus Mg content and (**b**) versus the inverse of Q.

Significant grain refinement can be achieved by increasing the Mg composition, as shown in Figure 5a by the substantial reduction in grain size due to the increase of solute Mg. By increasing the solute Mg content from 5% to 20%, a grain size reduction of approximately 2 mm is achieved, or around a 70% decrease.

The effectiveness of UST grain refinement is much more significant as shown in Figure 5a, producing grain sizes of about 120 μm or less, which is consistent with previous studies [33]. Not only is the grain refinement much more efficient, but there seems to be little in the way of dependence on solute content, with a variation of less than 50 μm between the alloys.

The initial addition of solute Mg was found to have a significant impact on the grain structure, compared to pure Al samples, which were found to have an average grain size of 280 μm when UST was applied. 

Both the constants *a* and *b* change as UST is applied, which can be clearly observed in Figure 5b. The difference in intercept appears to be small, however, when converting these values into grain densities (assuming spherical grains), it is found these correspond to an increase from 2436.7 grain/mm^3^ in the as-cast form, to 3933.1 grains/mm^3^ when UST is applied. This increase in grain density shows that while the intercept appears to be small, there is a notable and significant increase to the fraction of nuclei that have become active during solidification. This is further shown by the vast difference in grain densities in the Al–5Mg samples, where UST increases the grain density from 0.1 grains/mm^3^, to almost 1200 grains/mm^3^, an increase of four orders of magnitude.

According to cavitation theory, the number density of nucleant particles should increase with the application of UST as a function of the UST intensity [37]. It has previously been shown for intensive melt shearing of molten Al–Mg alloys that an increase in the number of oxide particles from the breakup of the oxide films results in additional nucleation [38].

The most notable difference is the dramatic reduction in the gradient of the 1/*Q* plots in Figure 5b. UST results in a much smaller value of *b*, orders of magnitude smaller. This change represents the near elimination of the NFZ.

The Interdependence Theory shows that the magnitude of NFZ is influenced by a combination of alloy composition and the casting conditions [18]. As the changes in alloy composition are the same for the two datasets, it can be determined that the reduction in the NFZ is a result of the application of the ultrasonic field. Reviewing Equation (3) shows that the gradient of these plots is not only influenced by material properties, but also the thermal gradient in the melt through the term *z*, which is related to the incremental undercooling required for further nucleation being controlled by the thermal gradient in the melt. The application of UST to aluminum alloys has previously been shown to reduce the thermal gradient throughout the whole melt [32,39]. Since the experimental conditions between the previous research and this study are the same, it is highly likely the same reduction in temperature gradient has occurred. If *z* is equal to zero, Equation (3) predicts that NFZ would be zero. Thus, all of the available activatable particles defined by x_Sd_ could become active nucleants.

The cavitation effect has previously been discussed as the source of undercooling and reduction of the thermal gradient in the melt [22] where it is proposed that the collapse of cavitation bubbles results in significant undercooling of the melt in a localized area. The net effect of the simultaneous bubble collapses could lead to a reduction of the thermal gradient of the melt, and minor undercooling. Other studies into this effect found that acoustic streaming generated by the application of the ultrasonic field is more likely the reason for homogenization of the temperature throughout the melt [28,29,32]. It seems likely that the acoustic streaming generated by the application of UST reduces the temperature gradient towards zero, resulting in this decrease in *x_nfz_* [28,32,37].

It should also be noted, that while the gradient of the UST grain size data appears flat, on closer inspection there is still a slight slope. This suggests that increases in *Q* still influence the resultant grain structure, although the role of *Q* is much less significant when UST is applied. Due to the limited data points and slight scattering in the data, it is difficult to determine the magnitude of this influence.

## 4. Conclusions

From this comparative study of the effect of Mg composition on grain size obtained with and without UST, it was found that the grain size dramatically decreased when UST was applied to all of the Mg compositions investigated. In addition, the dependence of grain size on the solute content was almost eliminated compared to the effect of Mg without the application of UST. While *Q* appears to retain some minor influence on the grain size formed under UST, this is much less significant compared to the reduction in the size of the nucleation free zone. This results in a reasonably consistent grain size over the range of Al–Mg alloys tested, although it should be noted that some initial solute content is required to achieve this significant grain refinement.

It is suggested, for the conditions used in this study and proposed in other studies [33,39], that the temperature gradient is reduced to very low values by UST-generated acoustic streaming in the melt, which, in turn, is the likely cause of a reduction in the size of the nucleation free zone.

## References

[1] Polmear I.J., StJohn D., Nie J.-F., Qian M. (2017). Light Alloys: Metallurgy of the Light Metals/Ian Polmear.

[2] Birru A.K., Karunakar D.B., Mahapatra M.M. (2012). A Study on hot tearing susceptibility of Al–Cu, Al–Mg, and Al–Zn alloys. Trans. Indian Inst. Met..

[3] Nagaumi H., Tsuchiya K. (2002). Effects of Mg contents on porosity formation in Al-Mg alloy DC slabs. J. Jpn. Inst. Light Met..

[4] Zolotorevsky V.S., Belov N.A., Glazoff M.V. (2007). Casting Aluminum Alloys.

[5] Hall E.O. (1951). The deformation and ageing of mild steel: III Discussion of results. Proc. Phys. Soc. Sect. B.

[6] Petch N.J.J. (1953). The Cleavage Strength of Polycristals. J. Iron Steel Inst..

[7] Jeong G., Park J., Nam S., Shin S.E., Shin J., Bae D., Choi H. (2015). The effect of grain size on the mechanical properties of aluminum. Arch. Metall. Mater..

[8] Lee Y.C., Dahle A.K., StJohn D.H. (2000). Grain Refinement of Magnesium. Proceedings of the Magnesium Technology 2000.

[9] Easton M., Wang H., Grandfield J., StJohn D., Sweet E. (2004). An analysis of the effect of grain refinement on the hot tearing of aluminium alloys. Materials Forum.

[10] Rana R.S., Purohit R., Das S. (2012). Reviews on the influences of alloying elements on the microstructure and mechanical properties of aluminum alloys and aluminum alloy composites. Int. J. Sci. Res. Publ..

[11] Ravi K.R., Manivannan S., Phanikumar G., Murty B.S., Sundarraj S. (2011). Influence of Mg on grain refinement of near eutectic Al-Si alloys. Metall. Mater. Trans. A.

[12] Rohatgi S., Tiwari M., Rathi A., Sharma A. (2015). Role of undercooling and effect of solute particles on grain refinement of aluminium alloys. Indian Foundry J..

[13] Buraś J., Szucki M., Piwowarski G., Krajewski W.K., Krajewski P.K. (2017). Strength properties examination of high zinc aluminium alloys inoculated with Ti addition. China Foundry.

[14] Chai G., Backerud L., Arnberg L. (1995). Relation between grain size and coherency parameters in aluminium alloys. Mater. Sci. Technol..

[15] Chen Z., He Z., Jie W. (2007). Model for evaluation of grain sizes of aluminum alloys with grain refinement additions. J. Mater. Sci. Technol..

[16] StJohn D.H., Easton M.A., Cao P., Qian M. (2007). New approach to analysis of grain refinement. Int. J. Cast Met. Res..

[17] Kozlov A., Schmid-Fetzer R. (2012). Growth restriction factor in Al-Si-Mg-Cu alloys. IOP Conf. Ser. Mater. Sci. Eng..

[18] StJohn D.H., Qian M., Easton M.A., Cao P. (2011). The interdependence theory: The relationship between grain formation and nucleant selection. Acta Mater..

[19] McCartney D.G. (1989). Grain refining of aluminium and its alloys using inoculants. Int. Mater. Rev..

[20] Sreekumar V.M., Eskin D.G. (2016). A new Al-Zr-Ti master alloy for ultrasonic grain refinement of wrought and foundry aluminum alloys. JOM.

[21] Murty B.S., Kori S.A., Chakraborty M. (2002). Grain refinement of aluminium and its alloys by heterogeneous nucleation and alloying. Int. Mater. Rev..

[22] Ėskin G.I. (1988). Ultrasonic Treatment of Light Alloy Melts.

[23] Song C., Han Q., Zhai Q. (2009). Review of grain refinement methods for as-cast microstructure of magnesium alloy. Chin. Foundry.

[24] Zhang L., Eskin D.G., Katgerman L. (2011). Influence of ultrasonic melt treatment on the formation of primary intermetallics and related grain refinement in aluminum alloys. J. Mater. Sci..

[25] Tuan N.Q., Puga H., Barbosa J., Pinto A.M.P. (2015). Grain refinement of Al-Mg-Sc alloy by ultrasonic treatment. Met. Mater. Int..

[26] Jiang R.P., Li X.Q., Zhang M. (2015). Investigation on the mechanism of grain refinement in aluminum alloy solidified under ultrasonic vibration. Met. Mater. Int..

[27] Wang G., Wang E.Q., Prasad A., Dargusch M., StJohn D.H., Murat T., Mark J., Glenn B. (2016). Grain Refinement of Al-Si Hypoeutectic Alloys by Al_3_Ti_1_B Master Alloy and Ultrasonic Treatment. Shape Casting: 6th International Symposium.

[28] Wang G., Dargusch M.S., Qian M., Eskin D.G., StJohn D.H. (2014). The role of ultrasonic treatment in refining the as-cast grain structure during the solidification of an Al–2Cu alloy. J. Cryst. Growth.

[29] Li J.W., Momono T., Fu Y., Jia Z., Tayu Y. (2007). Effect of ultrasonic stirring on temperature distribution and grain refinement in Al-1.65%Si alloy melt. Trans. Nonferrous Met. Soc. China.

[30] Eskin D.G. (2017). Ultrasonic processing of molten and solidifying aluminium alloys: Overview and outlook. Mater. Sci. Technol..

[31] Wang G., Croaker P., Dargusch M., McGuckin D., StJohn D. (2017). Simulation of convective flow and thermal conditions during ultrasonic treatment of an Al-2Cu alloy. Comput. Mater. Sci..

[32] Wang G., Dargusch M.S., Eskin D.G., StJohn D.H. (2017). Identifying the stages during ultrasonic processing that reduce the grain size of aluminum with added Al_3_Ti_1_B master alloy. Adv. Eng. Mater..

[33] Khalifa W., Tsunekawa Y., Okumiya M. (2008). Effect of ultrasonic melt treatment on microstructure of A356 aluminium cast alloys. Int. J. Cast Met. Res..

[34] Bhingole P.P., Chaudhari G.P. (2012). Synergy of nano carbon black inoculation and high intensity ultrasonic processing in cast magnesium alloys. Mater. Sci. Eng. A.

[35] Ramirez A., Qian M., Davis B., Wilks T., StJohn D.H. (2008). Potency of high-intensity ultrasonic treatment for grain refinement of magnesium alloys. Scr. Mater..

[36] (2010). ASTM E112-10, Standard Test Methods for Determining Average Grain Size, ASTM International: West Conshohocken, PA. http://www.astm.org/cgi-bin/resolver.cgi?E112.

[37] StJohn D.H., Easton M.A., Qian M., Taylor J.A. (2013). Grain refinement of magnesium alloys: A review of recent research, theoretical developments, and their application. Metall. Mater. Trans. A.

[38] Kumar S., Hari-Babu N., Scamans G.M., Fan Z. (2011). Influence of intensive melt shearing on the microstructure and mechanical properties of an Al-Mg alloy with high added impurity content. Metall. Mater. Trans. A.

[39] Ishiwata Y., Komarov S., Takeda Y., Hasso W., Anthony D.R., William A.C. (2012). Investigation of Acoustic Streaming in Aluminum Melts Exposed to High-Intensity Ultrasonic Irradiation. ICAA13 Pittsburgh.

